# One‐Year Outcomes of Short‐Term Dual Antiplatelet Therapy Following Percutaneous Coronary Intervention With Drug‐Eluting Stents: A Meta‐Analysis of Randomized Clinical Trials

**DOI:** 10.1002/ccd.70163

**Published:** 2025-09-17

**Authors:** Thomas Fretz, Srikiran Dasari, John Sakaleros, Abdul Mueez Alam Kayani, Nathaniel Bluckner, Kristina Pond, Nathan Markus, Alejandra Cardona‐Perez, Alan Garcia, Ricky Lemus‐Zamora, Jeffrey Breall

**Affiliations:** ^1^ Division of Internal Medicine Indiana University School of Medicine Indianapolis Indiana USA; ^2^ AdventHealth Tampa Tampa Florida USA; ^3^ Division of Cardiovascular Medicine Indiana University School of Medicine Indianapolis Indiana USA

**Keywords:** drug‐eluting stent, dual antiplatelet therapy, percutaneous coronary intervention

## Abstract

Dual antiplatelet therapy (DAPT) is recommended after percutaneous coronary intervention (PCI), though the optimal duration is unclear. DAPT reduces stent thrombosis, repeat myocardial infarction, and cardiovascular death, though at the cost of increased bleeding events. Currently, both European and American guidelines recommend a 6‐month duration of DAPT following PCI with drug‐eluting stents (DES) for stable coronary disease and a 12‐month regimen following PCI for acute coronary syndrome. Recent randomized clinical trials (RCTs) suggest a shorter duration of DAPT may be acceptable. PubMed, EMBASE, and Cochrane databases were queried from inception to June 2025 to identify RCTs comparing short ( ≤ 3 months) with traditional durations of DAPT following PCI with DES and reporting outcomes of interest at 1 year, including major adverse cardiovascular and cerebrovascular events (MACCE) and net adverse clinical events (NACE). Individual endpoints including mortality, cardiovascular mortality, myocardial infarction, stroke, stent thrombosis, significant bleeding, and target vessel revascularization were analyzed. Effect estimates were pooled using a random‐effects model and reported as risk ratios (RR) for dichotomous outcomes with 95% confidence intervals. Thirteen studies met the inclusion criteria, reporting results on 53,421 patients, of whom 26,712 patients were in the short DAPT cohort and 26,719 in the traditional DAPT cohort. Duration of DAPT ranged from 1 to 3 months. Ten studies used P2Y12 inhibitors as the single antiplatelet agent following DAPT, whereas three studies used aspirin. Patients were 76.0% male, mean age 64.0 years, and 64.9% with ACS on presentation. Shorter duration of DAPT significantly decreased NACE (RR: 0.80; [0.71, 0.91], *p* < 0.001) without impacting MACE (RR: 0.98; [0.89, 1.07], *p* = 0.64) at 1 year following PCI with DES. A 3‐month duration of DAPT demonstrated favorable results over shorter durations, and monotherapy with a high‐potency P2Y12 inhibitor was preferable over aspirin or a low‐potency P2Y12 inhibitor. In patients who underwent a PCI with DES placement, a 3‐month duration of DAPT decreased NACE without impacting other MACCE compared to guideline‐directed DAPT durations.

**Abbreviations**
ACS: acute coronary syndrome
BARC: Bleeding Academic Research Consortium
DAPT: dual antiplatelet therapy
DES: drug‐eluting stent
MACCE: major adverse cardiovascular and cerebrovascular events
NACE: net adverse clinical events
PCI: percutaneous coronary intervention
RCT: randomized clinical trials
SAPT: single antiplatelet therapy

## 1 Introduction

Dual antiplatelet therapy (DAPT) with aspirin and a P2Y12 inhibitor is the cornerstone of medical therapy following percutaneous coronary intervention (PCI). DAPT reduces recurrent ischemic events, most notably in‐stent thrombosis, the risks of which are markedly elevated following PCI [1]. With appropriate treatment, the risk of ischemic events generally decreases over the 1−3 months after intervention, whereas the risk of bleeding persists and is proportional to the intensity and duration of treatment [2]. The reductions of these ischemic events like, stent thrombosis and myocardial infarction, must be weighed against the increased bleeding risks associated with these DAPT, as both recurrent ischemia and bleeding are associated with significantly increased morbidity and mortality [3, 4]. Weighing these risks, both US [5] and European [6] guidelines currently recommend 6‐ and 12‐month durations of DAPT following PCI with drug‐eluting stents (DES) for stable coronary artery disease and acute coronary syndrome (ACS), respectively, in patients without high bleeding risk, followed by single antiplatelet therapy (SAPT) indefinitely [7].

The risk‐benefit equation of DAPT after PCI has evolved, however, with the development of new medical and interventional therapies, like more potent P2Y12 inhibitors and newer generation DES [8, 9]. Recent randomized clinical trials (RCTs) have brought into question the appropriate duration of DAPT following PCI and suggest it may be shorter than current recommendations. Despite these trials, multiple questions remain unclear, such as the optimal duration of DAPT after PCI, the choice of agent in SAPT, and which specific populations may benefit most from shorter durations of DAPT. To answer these questions, we conducted a meta‐analysis of RCTs examining a shorter duration of DAPT.

## 2 Methods

### 2.1 Study Design

This meta‐analysis was conducted in accordance with the guidelines of Cochrane's Preferred Reporting Items for Systematic Reviews and Meta‐Analyses [10] (Supporting Information S1: S1). The study was exempt from institutional review board approval since only published, publicly available data was used. This study was prospectively registered with PROSPERO, an international systematic review registry (ID 1065336).

### 2.2 Data Sources and Search Strategy

A literature search of PubMed, Embase, and the Cochrane Collaboration Central Register of Controlled Trials was conducted from inception until March 2025 for RCTs comparing different durations of DAPT following PCI. The search strategy is shown in Supporting Information S1: S2. Two authors independently screened articles for inclusion, retrieved potentially relevant studies, and determined their eligibility. Disagreements were resolved by consensus. No restrictions were set on language.

### 2.3 Eligibility Criteria and End Points

Studies were considered if they fulfilled the following criteria: (1) are published or presented RCTs; (2) enrolled patients who underwent PCI with DES; (3) compared different durations of DAPT with one arm receiving a short course ( ≤ 3 months) DAPT and another arm receiving guideline‐based (6−12 months) durations; and (4) reported outcomes of interest. We set no restrictions for date or language. The HOST‐BR trial, presented at the 2025 American College of Cardiology meeting, was included for completeness.

Data was extracted from publicly available records using a standardized data collection software. Information such as study year, author, protocol, and other general information was collected. Patient demographic and relevant comorbidity data were collected. When outcome data were not available from the original published article, other publications based on the same trial were queried.

The co‐primary endpoints were (1) major adverse cardiovascular or cerebrovascular events (MACCE) and (2) net adverse clinical events (NACE). MACCE was generally defined as a composite of mortality, nonfatal myocardial infarction, and nonfatal stroke. NACE was a composite of MACCE and major bleeding events. MACCE and NACE were decided on a case‐by‐case basis if these specific endpoints varied when reported by included studies. The endpoints were examined at 1 year following PCI. Secondary endpoints measured in this analysis included: all‐cause mortality, cardiovascular mortality, myocardial infarction, definite or probable stent thrombosis, bleeding, and target vessel revascularization. We considered only Bleeding Academic Research Consortium (BARC) types 3 or 5 when available.

### 2.4 Quality Assessment

A risk of bias assessment was independently performed for each trial by two authors using the Cochrane Risk of Bias Tool. An additional crossover bias was defined as more than 20% in‐study crossover in at least one study arm. Publication bias assessment was performed using a funnel plot when 10 or more studies reported on an outcome.

### 2.5 Statistical Analysis

For descriptive analyses, categorical data were expressed as percentages. Continuous data were expressed as reported in individual studies with both means and standard deviations. Summary statistics for continuous data are expressed as means after converting medians and quartiles using standardized algorithms [11].

Meta‐analysis was performed with a random‐effects model (Der Simonian and Laird's approach) using Review Manager, version 5.4 (Cochrane Collaboration, Oxford, United Kingdom). Outcomes of interest were reported as a risk ratio (RR) with 95% confidence interval (CI) using an inverse variance random effects model. A *p* ≤ 0.05 was considered to be statistically significant. The heterogeneity of effect size estimates across studies was quantified using the Higgins *I* ^2^ statistic. Heterogeneity was classified as “low” if *I* ^2^ was less than 20%, “moderate” if between 20% and 50%, and “high” if heterogeneity was greater than 50% [12]. For each outcome, a sensitivity analysis was performed using the one‐out approach, where one study is sequentially removed to assess the impact on the overall outcomes to determine if any single study disproportionately affects the results.

Subgroup analysis was performed to compare the impact of other factors on the endpoints including class of antiplatelet monotherapy following short DAPT, high and low potency P2Y12 inhibitors, and 1‐month versus 3‐month durations of DAPT. An additional analysis of exclusively patients presenting with ACS was performed, with a similar subgroup analysis within this cohort.

## 3 Results

### 3.1 Study Characteristics

Thirteen studies [13, 14, 15, 16, 17, 18, 19, 20, 21, 22, 23, 24, 25] met the inclusion criteria comprising 53,421 patients, of whom 26,712 patients were in the short DAPT cohort and 26,719 in the traditional DAPT cohort. The flowchart of study selection is shown in Supporting Information S1: S4. The duration of DAPT ranged from 1 to 3 months. Outcomes of interest were obtained for all studies at 12 months, though two studies reported 2‐year outcomes [14, 16]. Three studies reported outcomes of interest in separate publications [26, 27, 28]. Ten studies used P2Y12 inhibitors as monotherapy following short DAPT; three studies used aspirin. The P2Y12 inhibitors are ticagrelor, clopidogrel, and prasugrel. Patients were 76.0% male, mean age 64.0 years, and 64.9% with ACS on presentation.

All studies reported MACCE. This composite outcome varied slightly between studies, though all included mortality, myocardial infarction, and stroke, with some including stent thrombosis and target vessel revascularization. All studies except TWILIGHT reported NACE, which was generally a composite of MACCE and bleeding outcomes. All studies except TWILIGHT included patients without increased bleeding risks which this trial defined based on both clinical and angiographic criteria. A brief summary of the included trials, patient characteristics, and definitions for MACCE and NACE are included in Table 1.

**Table 1:** Study design and patient characteristics.

Study year	Size	ACS (%)	Male (%)	Age (years)	High bleeding risk?	Length, short DAPT	Monotherapy agent	Study design	Location	MACCE	NACE
RESET 2012	2117	55	64	62	No	3 months	Aspirin	Open label	South Korea	Mortality, MI, ST	CV mortality, MI, TVR, bleeding
OPTIMIZE 2013	3119	32	63	62	No	3 months	Aspirin	Open label	Brazil	Mortality, MI, TVR, urgent CABG	Mortality, MI, CVA, bleeding
GLOBAL LEADERS 2019	15,968	47	77	65	No	1 month	Ticagrelor	Open label	Europe, Australia, Brazil, Canada, Singapore	Mortality, MI, CVA	Mortality, MI, CVA, bleeding
SMART CHOICE 2019	2993	58	73	65	No	3 months	Clopidogrel (77%); other P2Y12i (23%)	Open label	South Korea	Mortality, MI, CVA	Mortality, MI, CVA, bleeding
REDUCE 2019	1460	100	79	61	No	3 months	Aspirin	Open label	Europe, India, Singapore, Malaysia, Hong Kong	Mortality, ST, TVR, CVA	Mortality, ST, TVR, CVA, bleeding
TWILIGHT 2020	7119	65	76	65	Yes (clinical + angiographic RF)	3 months	Ticagrelor	Double blind	Europe, North America, China, India, Israel	Mortality, MI, CVA	NR
TICO 2020	3056	100	79	61	No	3 months	Ticagrelor	Open label	South Korea	Mortality, MI, ST, TVR, CVA	Mortality, MI, ST, TVR, CVA, bleeding
STOPDAPT‐2 2022	5997	69	78	68	No	1 month	Clopidogrel	Open label	Japan	CV morality, MI, ST, CVA	CV morality, MI, ST, CVA, bleeding
T‐PASS 2023	2850	100	83	61	No	< 1 month	Ticagrelor	Open label	South Korea	Mortality, MI, CVA	Mortality, MI, CVA, bleeding
ULTIMATE DAPT 2024	3400	100	74	63	No	1 month	Ticagrelor	Double blind	UK, China, Italy, Pakistan	CV mortality, MI, ST, TVR, CVA	CV mortality, MI, ST, TVR, CVA, bleeding
SHARE 2024	1387	74	76	63	No	3 months	Clopidogrel (62%); ticagrelor (38%)	Open label	South Korea	CV mortality, MI, ST, TVR, CVA	CV mortality, MI, ST, TVR, CVA, bleeding
HOST BR 2025	3299	63	79	63	No	3 months	Clopidogrel	Open label	South Korea	CV mortality, MI, ST, CVA	CV mortality, MI, ST, CVA, bleeding
4D‐ACS 2025	656	100	83	61	No	1 month	Pasugrel	Open label	South Korea	CV mortality, MI, TVR, CVA	Mortality, MI, TVR, CVA, bleeding

Abbreviations: ACS = acute coronary syndrome, CABG = coronary artery bypass graft surgery, CV = cardiovascular, CVA= cerebrovascular accident, DAPT = dual antiplatelet therapy, MACCE = major adverse cardiovascular and cerebrovascular events, MI = myocardial infarction, NACE = net adverse clinical events, P2Y12i = P2Y12 inhibitor, RF = risk factor, ST = stent thrombosis, TVR = target lesion revascularization, UK = United Kingdom.

### 3.2 Quality Assessment

The studies were generally of low risk for standard measures of bias, except for blinding of participants and personnel. The majority of studies were open‐label and non‐placebo controlled which introduced bias in that regard, though potentially compensated for this by blinding of outcome assessment. The Cochrane Risk of Bias Tool is shown in Supporting Information S1: S5. No outcome evaluated by funnel plot was noted to be indicative of publication bias as each was relatively symmetric on visual inspection.

## 4 Entire Cohort Analysis

### 4.1 Primary Analysis

**Figure 1:**
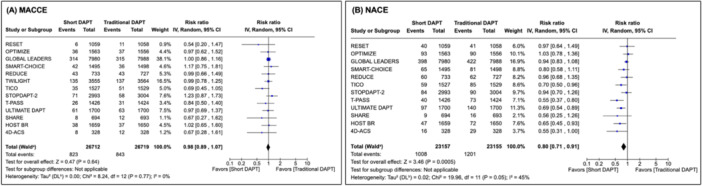
Forest plot of primary endpoints for entire cohort: MACCE (A), NACE (B). This figure reports MACCE and NACE in traditional and short DAPT cohorts across the included studies as risk ratios with 95% confidence intervals and an inverse variance random effects model. *p* < 0.05 denotes a statistically significant result. No difference was observed between traditional and short DAPT with respect to MACCE; however, NACE were significantly lower in the short DAPT group. Heterogeneity quantified by Higgins *I* ^2^ statistics is low in the MACCE group and moderate in the NACE group. DAPT = dual antiplatelet therapy, MACCE = major adverse cardiovascular or cerebrovascular events, NACE = net adverse clinical events. [Color figure can be viewed at wileyonlinelibrary.com]

All outcomes were analyzed at 1 year for all patients who underwent PCI with DES which includes ACS and chronic coronary disease indications. There was no difference observed between short DAPT and traditional duration of DAPT with respect to the co‐primary endpoint MACCE (RR: 0.98, CI: [0.89−1.07], *p* = 0.64; *I* ^2^ = 0%). There was a statistically significant reduction in the co‐primary endpoint NACE in patients treated with short DAPT compared to traditional durations (RR: 0.80, CI: [0.71−0.91], *p* < 0.001; *I* ^2^ = 45%) (Figure 1). Among secondary endpoints, only major bleeding reached statistical difference between short and traditional DAPT (RR: 0.51, CI: [0.39−0.66], *p* < 0.001; *I* ^2^ = 57%). No other individual endpoint reached a statistically significant difference between short and traditional DAPT. Endpoints such as mortality (RR: 0.97, *p* = 0.69), cardiovascular mortality (RR: 0.86, *p* = 0.20), target lesion revascularization (RR: 0.98, *p* = 0.71), and stroke (RR: 0.96, *p* = 0.72) favored short DAPT, whereas myocardial infarction (RR: 1.09, *p* = 0.24) and stent thrombosis (RR: 1.14, *p* = 0.35) numerically favored traditional DAPT. Forest plots and complete results for individual endpoints are shown in Supporting Information S1: S6.

### 4.2 Subgroup Analysis

Subgroup analyses of the total cohort were performed. The first subgroup analysis compared monotherapy classes after completion of short DAPT. All trials used either a P2Y12 inhibitor or aspirin for monotherapy. There was no difference between short DAPT followed by P2Y12 inhibitor monotherapy (RR: 0.99, *p* = 0.82) or aspirin monotherapy (RR: 0.93, *p* = 0.64) compared to traditional DAPT durations with respect to MACCE. Monotherapy with P2Y12 inhibitors demonstrated a statistically significant reduction in NACE (RR: 0.75, *p* < 0.001) compared to traditional DAPT, whereas monotherapy with aspirin (RR: 0.99, *p* = 0.96) did not show such a reduction, and this difference between classes was statistically significant (*p* = 0.02). Only P2Y12 inhibitor monotherapy demonstrated a statistically significant reduction in major bleeding (RR: 0.47, *p* < 0.001). Traditional DAPT was numerically favored without reaching statistical significance in all other individual endpoints besides major bleeding compared to short DAPT with aspirin monotherapy.

Trials were grouped by the potency of the P2Y12 inhibitor used for monotherapy following short DAPT. Both high‐potency and low‐potency P2Y12 inhibitors demonstrated statistically significant reduced NACE and major bleeding events compared to traditional durations of DAPT. Only short DAPT followed by high potency P2Y12 inhibitor decreased overall mortality (RR: 0.83, *p* = 0.05). The high‐potency P2Y12 inhibitor had numerically favorable MACCE outcomes compared to traditional durations of DAPT, as well as all individual endpoints except for myocardial infarction (RR: 1.03, *p* = 0.41) and stent thrombosis (RR: 1.13, *p* = 0.46). The low‐potency P2Y12 inhibitor cohort demonstrated numerically higher MACCE (RR: 1.11, *p* = 0.35), overall mortality (RR: 1.29, *p* = 0.12), and several individual endpoints compared to traditional DAPT duration.

The trials were also analyzed by duration of short DAPT, grouped into a 3‐month cohort and 1‐month cohort. Neither cohort demonstrated a statistically significant difference compared to traditional durations of DAPT with respect to MACCE. The 3‐month cohort significantly reduced NACE (RR: 0.79, *p* = 0.005) and major bleeding events (RR: 0.52, *p* < 0.001) compared to traditional duration DAPT. MACCE and the other individual endpoints numerically favored the 3‐month duration of DAPT, though did not reach statistical significance. While 1‐month cohort reached statistical significance in reducing major bleeding (RR: 0.43, CI: [0.22−0.86], *p* = 0.02; *I* ^2^ = 80%) compared to traditional durations, no other outcome reached significance. Traditional DAPT duration was favored over 1‐month DAPT with respect to MACCE, mortality, myocardial infarction, stent thrombosis, and target vessel revascularization, though this did not reach statistical significance.

The pooled results of the primary analysis and subgroup analysis involving the entire cohort are demonstrated in Table 2. Individual forest plots are provided in Supporting Information S1: S5–S11.

**Table 2:** Pooled risk ratios and 95% confidence intervals compared to traditional duration of DAPT.

Entire cohort Pooled analysis by subgroup compared to guideline‐based DAPT duration RR (95% CI)							
Outcome	Entire cohort	Short DAPT followed by any P2Y12i monotherapy	Short DAPT followed by aspirin monotherapy	Short DAPT followed by HP P2Y12i monotherapy	Short DAPT followed by LP P2Y12i monotherapy	1 month DAPT followed by P2Y12i monotherapy	3 month DAPT followed by P2Y12i monotherapy
MACCE	0.98 (0.89–1.07)	0.98 (0.89–1.09)	0.93 (0.70–1.25)	0.95 (0.85–1.07)	1.11 (0.89–1.39)	1.01 (0.89–1.15)	0.94 (0.80–1.10)
NACE	0.80 (0.71–0.91)	0.75 (0.64–0.87)	0.99 (0.82–1.21)	0.71 (0.56–0.90)	0.79 (0.66–0.95)	0.82 (0.67–1.01)	0.67 (0.57–0.80)
Mortality	0.97 (0.82–1.15)	0.93 (0.79–1.10)	1.25 (0.66–2.40)	0.83 (0.68–1.00)	1.29 (0.94–1.78)	1.06 (0.72–1.56)	0.90 (0.69–1.17)
CV mortality	0.86 (0.69–1.08)	0.83 (0.64–1.07)	1.05 (0.51–2.14)	0.74 (0.51–1.08)	0.92 (0.64–1.31)	1.04 (0.60–1.80)	0.78 (0.58–1.04)
MI	1.09 (0.95–1.24)	1.07 (0.89–1.28)	1.13 (0.80–1.60)	1.07 (0.91–1.26)	1.01 (0.56–1.81)	1.28 (0.95 ‐ 1.74)	0.90 (0.71–1.14)
Stent thrombosis	1.14 (0.87–1.48)	1.15 (0.86–1.53)	1.12 (0.43–2.91)	1.13 (0.82–1.56)	1.26 (0.63–2.52)	1.37 (0.95–1.97)	0.87 (0.55–1.38)
TVR	0.98 (0.87–1.10)	0.95 (0.81–1.11)	1.14 (0.89–1.47)	0.87 (0.76–1.01)	1.22 (0.90–1.66)	1.03 (0.78–1.34)	0.82 (0.56–1.19)
Stroke	0.98 (0.80–1.20)	0.98 (0.77–1.25)	1.05 (0.35–3.18)	0.97 (0.70–1.35)	1.00 (0.64–1.56)	0.96 (0.72–1.28)	0.98 (0.77–1.25)
Major bleeding	0.51 (0.39–0.66)	0.49 (0.36–0.67)	0.72 (0.45–1.15)	0.49 (0.33–0.72)	0.43 (0.26–0.71)	0.43 (0.22–0.86)	0.52 (0.41–0.65)

*Note:* Dark blue is statistically significant in favor of the experimental DAPT cohort; light blue favors the experimental DAPT cohort without statistical significance; light red favors the traditional DAPT cohort; dark red is statistically significant in favor of the traditional DAPT cohort.

Abbreviations: CV = cardiovascular, DAPT = dual antiplatelet therapy, HP = high potency, LP = low potency, MACCE = major adverse cardiovascular and cerebrovascular events, MI = myocardial infarction, NACE = net adverse clinical events, TVR = target vessel revascularization.

## 5 ACS Subgroup Analysis

### 5.1 Primary Analysis

**Figure 2:**
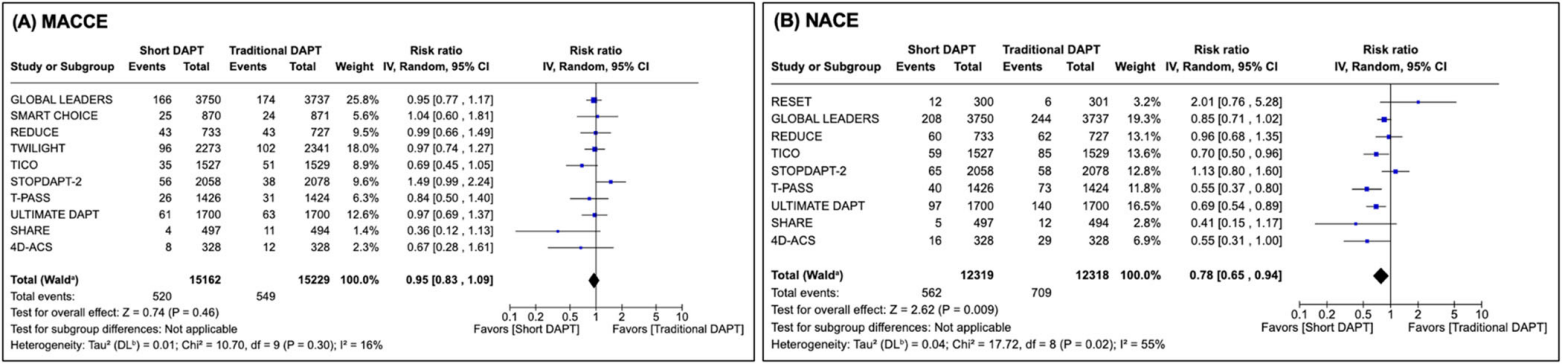
Forest plot of primary endpoints for ACS cohort: MACCE (A), NACE (B). This figure reports MACCE and NACE across ACS subgroups in traditional and short DAPT cohorts as risk ratios with 95% confidence intervals and an inverse variance random effects model. *p* < 0.05 denotes a statistically significant result. No difference was observed between traditional and short DAPT with respect to MACCE in ACS patients; however, NACE were significantly lower in the short DAPT group in ACS patients. Heterogeneity quantified by Higgins *I* ^2^ statistics is moderate in the MACCE group and high in the NACE group. ACS = acute coronary syndrome, DAPT = dual antiplatelet therapy, MACCE = major adverse cardiovascular or cerebrovascular events, NACE = net adverse clinical events. [Color figure can be viewed at wileyonlinelibrary.com]

All outcomes were analyzed at 1 year for all patients who underwent PCI with DES for ACS. A total of 30,391 patients with ACS were included in this subgroup analysis, with 15,162 in the short DAPT cohort and 15,229 in the traditional DAPT cohort. Amongst all patients with ACS who underwent PCI with DES, a shorter duration of DAPT was not significantly different compared to traditional duration with respect to MACCE (RR: 0.95, CI: [0.83−1.09], *p* = 0.46; *I* ^2^ = 16%). There was a statistically significant reduction in NACE amongst the short DAPT cohort compared to the traditional DAPT cohort (RR: 0.78, CI: [0.65−0.94], *p* = 0.009; *I* ^2^ = 55%) (Figure 2). Amongst individual endpoints, only major bleeding reached statistical significance, with a reduction amongst the short DAPT cohort (RR: 0.50, CI: [0.39−0.65], *p* < 0.001; *I* ^2^ = 43%). Overall mortality, cardiovascular mortality, target vessel revascularization, and stroke numerically favored short DAPT durations, whereas myocardial infarction and stent thrombosis numerically favored traditional DAPT durations, though these outcomes did not reach statistical significance.

### 5.2 Subgroup Analysis

A subgroup analysis was performed in a similar manner to the entire cohort analysis. A short duration of DAPT followed by P2Y12 monotherapy significantly reduced NACE (RR: 0.73, *p* = 0.001) and numerically lowered MACCE compared to traditional durations of DAPT, though this did not reach statistical significance (RR: 0.94, *p* = 0.45). There was no statistical significance between traditional DAPT and short DAPT followed by aspirin monotherapy in any measured outcomes.

A regimen of short DAPT followed by high‐potency P2Y12 inhibitors significantly reduced NACE (RR: 0.71, *p* < 0.001), overall mortality (RR: 0.80, *p* = 0.05), and major bleeding (RR: 0.45, *p* < 0.001) without impacting MACCE (RR: 0.91, *p* = 0.18). A short duration of DAPT followed by low potency P2Y12 inhibitors demonstrated a statistically significant increase in myocardial infarctions (RR: 1.90, *p* = 0.03) compared to traditional DAPT. This cohort did not differ significantly from traditional DAPT duration with other outcomes.

Both 1‐month and 3‐month DAPT followed by P2Y12 inhibitor had generally favorable results. These cohorts significantly decreased both NACE and major bleeding without impacting MACCE. Though not statistically significant, a 3‐month DAPT with P2Y12 inhibitor monotherapy had numerically lower MACE, overall mortality, cardiovascular mortality, myocardial infarction, stent thrombosis, and target vessel revascularization. The 1 month duration demonstrated increased rates of myocardial infarction and stent thrombosis, though this did not reach statistical significance.

Pooled results of the ACS cohort analysis, including subgroup analysis, are shown in Table 3. Individual forest plots are provided in Supporting Information S1: S12–18.

**Table 3:** Pooled risk ratios and 95% confidence intervals compared to traditional duration of DAPT in the acute coronary syndrome cohort.

ACS cohort Pooled analysis by subgroup compared to guideline‐based DAPT duration RR (95% CI)							
Outcome	Entire cohort	Short DAPT followed by any P2Y12i monotherapy	Short DAPT followed by aspirin monotherapy	Short DAPT followed by HP P2Y12i monotherapy	Short DAPT followed by LP P2Y12i monotherapy	1 month DAPT followed by P2Y12i monotherapy	3 month DAPT followed by P2Y12i monotherapy
MACCE	0.95 (0.83–1.09)	0.94 (0.81–1.10)	0.99 (0.66–1.49)	0.91 (0.80–1.04)	1.00 (0.55–1.83)	1.00 (0.83–1.21)	0.84 (0.63–1.10)
NACE	0.78 (0.65–0.94)	0.73 (0.61–0.89)	1.20 (0.62–2.33)	0.71 (0.60–0.84)	0.77 (0.30–2.01)	0.76 (0.60–0.95)	0.66 (0.49–0.90)
Mortality	0.97 (0.73–1.27)	0.88 (0.69–1.12)	2.36 (0.95–5.88)	0.80 (0.63–1.00)	1.49 (0.83–2.66)	1.00 (0.73–1.36)	0.68 (0.45–1.02)
CV mortality	0.96 (0.62–1.50)	0.85 (0.53–1.36)	2.64 (0.70–9.93)	0.79 (0.46–1.38)	1.01 (0.42–2.42)	0.97 (0.56–1.66)	0.58 (0.23–1.48)
MI	1.11 (0.91–1.36)	1.11 (0.87–1.41)	1.20 (0.60–2.42)	1.03 (0.85–1.26)	1.90 (1.06–3.41)	1.24 (0.91–1.69)	0.89 (0.56–1.42)
Stent thrombosis	1.15 (0.80–1.66)	1.08 (0.73–1.58)	1.66 (0.25–11.05)	0.97 (0.64–1.45)	2.52 (0.79–8.04)	1.22 (0.77–1.92)	0.84 (0.34–2.03)
TVR	0.97 (0.77–1.23)	0.93 (0.75–1.15)	1.75 (0.40–7.76)	0.85 (0.70–1.02)	1.45 (0.93–2.27)	0.95 (0.74–1.22)	0.80 (0.32–2.02)
Stroke	0.98 (0.74–1.30)	0.99 (0.74–1.30)	0.66 (0.11–3.95)	0.92 (0.67–1.25)	1.41 (0.73–2.73)	0.99 (0.71–1.36)	1.02 (0.53–1.95)
Major bleeding	0.50 (0.39–0.65)	0.48 (0.39–0.65)	0.81 (0.44–1.50)	0.45 (0.33–0.61)	0.60 (0.29–1.28)	0.43 (0.29–0.63)	0.54 (0.36–0.87)

*Note:* Dark blue is statistically significant in favor of the experimental DAPT cohort; light blue favors the experimental DAPT cohort without statistical significance; light red favors the traditional DAPT cohort; dark red is statistically significant in favor of the traditional DAPT cohort.

Abbreviations: CV = cardiovascular, DAPT = dual antiplatelet therapy, HP = high potency, LP = low potency, MACCE = major adverse cardiovascular and cerebrovascular events, MI = myocardial infarction, NACE = net adverse clinical events, TVR = target vessel revascularization.

### 5.3 Sensitivity Analysis

Primary and secondary endpoints in the primary analysis were evaluated with the one‐out approach for sensitivity analysis to determine if removal of one study significantly affected heterogeneity and the overall result. Using this approach, there were no significant changes to the results in the primary analysis in either statistical significance or heterogeneity. A sensitivity analysis was performed in the same manner after removal of the TWILIGHT study, given its was the only study with patients at increased risks of bleeding. Removal of this study and sensitivity analysis with the one‐out approach again did not significantly alter the statistical significance of outcomes or alter heterogeneity.

## 6 Discussion

This systematic review and meta‐analysis of 13 RCTs including 53,421 patients undergoing PCI with DES compared clinical outcomes of short DAPT durations at 1 year. The main findings of the study are as follows: (1) A 3‐month duration DAPT followed by single antiplatelet monotherapy reduces NACE without impacting MACCE; (2) high potency P2Y12 inhibitors outperform low potency P2Y12 inhibitors and aspirin as monotherapy agents following a short duration of DAPT with respect to NACE, mortality, and individual ischemic endpoints; (3) a 3‐month duration of DAPT outperforms 1‐month duration of DAPT with respect to ischemic outcomes; (4) these results are consistent within an ACS population.

Determining the optimal duration of DAPT following PCI is critical for minimizing recurrent ischemic events while balancing bleeding risk. Recurrent stent thrombosis and bleeding while on DAPT are potentially devastating complications of PCI, and each is associated with a several‐fold increased risk of mortality [3, 4]. Percutaneous revascularization directly causes endothelial injury to the coronary artery. The inflammatory response and subsequent fibroblast proliferation, neointimal hyperplasia, and platelet adherence to the foreign body can lead to stent thrombosis [29]. DES are designed to release anti‐proliferative medications that modulate these proliferative and inflammatory reactions. Contemporary DES significantly improves outcomes like stent thrombosis and repeat myocardial infarctions over previous generations of stents, reducing rates of stent thrombosis, myocardial infarctions, and overall stent failure. More recent innovations, like ultra‐thin or bioresorbable stents, show promise in further reductions in stent failure [9, 30]. Medical therapy has also advanced significantly since the initial trials involving clopidogrel, which form the basis of current recommendations [31]. DAPT with aspirin and a P2Y12 inhibitor reduces platelet activation and consequently the risk of stent thrombosis, leading to decreased myocardial infarctions and revascularizations—though at the expense of increased bleeding [1, 32]. Newer P2Y12 inhibitors, ticagrelor and prasugrel, are more potent, have more predictable pharmacodynamics, and have superior cardiovascular outcomes compared to clopidogrel [33, 34]. In light of this evolution in both interventional and antiplatelet therapies require a new evaluation of best practices.

Currently, both US and European societies recommend a 6 month duration of DAPT for PCI of chronic coronary disease and a 12 month duration for those presenting with ACS and without high risk of bleeding [5, 6, 7], with exceptions made for those with increased risk of bleeding [35]. Previous meta‐analyses have focused on shorter durations of DAPT after PCI in patients with ACS, older adults, patients with high risk of bleeding, and those who receive P2Y12 monotherapy and have demonstrated similar outcomes as this analysis [36, 37, 38]. It is a nearly ubiquitous conclusion that decreasing the duration of DAPT decreases significant bleeding events without negatively impacting ischemic outcomes like stent thrombosis, myocardial infarction, and cardiovascular death. Some analyses have even suggested a mortality benefit to shorter DAPT [39, 40], though this has not been consistently demonstrated. This analysis is consistent with these previous works, though it is unique in its size and scope, with several clinically impactful findings. First, in both the entire cohort and the ACS subgroup, a 3‐month duration of DAPT showed favorable outcomes over traditional durations in nearly all measures. Second, amongst monotherapy agents following short DAPT, high potency P2Y12 inhibitors outperformed other monotherapy classes and demonstrated an overall mortality benefit in both the entire and ACS cohort. It is important to note that these results are limited to 1 year following PCI, and this analysis does not attempt to characterize events after this time. More long‐term studies are necessary to characterize this and determine optimal monotherapy after 1 year.

This contemporary analysis demonstrates a decreased duration of DAPT as an acceptable alternative to the current guidelines following PCI for both acute and chronic coronary syndromes. The most appropriate recommendation based on the evidence presented in this analysis is a 3‐month duration of DAPT followed by high‐intensity P2Y12 inhibitor in both acute and chronic coronary syndromes in patients with normal bleeding risk. A 1‐month duration of DAPT is potentially harmful with respect to myocardial infarction and stent thrombosis outcomes and may be too short a duration. This finding is generally consistent with previous research showing risk of recurrent ischemia is highest within the first 3 months after PCI [2], though it should be noted several included studies had conflicting results. These differences may be in part due to the use of high‐intensity P2Y12 inhibitors in some trials and clopidogrel in others. For example, the STOPDAPT‐2 ACS trial comparing 1−2 months of DAPT followed by clopidogrel monotherapy to 12 months in patients with ACS did not meet its non‐inferiority endpoints [41]. These are similar findings in the high bleeding risk stratum of the HOST‐BR study, which compared a 1−3 month duration of DAPT followed by clopidogrel monotherapy. The 1‐month DAPT cohort had significantly higher rates of target lesion revascularization, ischemic strokes, and cardiovascular death compared to the 3‐month counterparts. This contrasts to the GLOBAL LEADERS, T‐PASS, ULTIMATE‐DAPT trials that all found non‐inferiority of a 1‐month DAPT duration followed by ticagrelor monotherapy. This data is also consistent with the MASTER DAPT trial which found noninferiority of 1‐month DAPT compared to 3‐month DAPT in patients with high bleeding risk [42]. These findings support the recent updates to US guidelines to allow decreasing DAPT duration to 1 month followed by high intensity P2Y12 inhibitor therapy in patients with elevated bleeding risks [35]. More data is necessary before applying a 1‐month DAPT recommendation to patients with normal bleeding risk. On the contrary, increased durations of DAPT have been shown to reduce ischemic events [43] and it may be reasonable to continue DAPT longer than a 3 month duration to prevent ischemic events in a select group of patients with low bleeding risks and increased cardiovascular risks, though this decision should be made on a case‐by‐case basis after a discussion of the risks and benefits.

## 7 Limitations

Though this analysis has a large sample size, the study protocols, monotherapy choice, and patient populations were heterogeneous, which limits definitive conclusions. Within the ACS analysis, the low‐potency P2Y12 inhibitor and aspirin subgroup analysis were limited due to the number of studies. This analysis is also limited in that follow‐up was limited to 12 months, which may favor shorter durations of DAPT, given that a significant portion of stent failure occurs after 12 months. In a large meta‐analysis, very late stent thrombosis, defined as greater than 1 year, accounted for about one quarter of all stent thrombosis [44], though some analysis suggests very late stent thrombosis is most often due to stent malposition [45]. It is not excluded from our analysis that shorter durations of DAPT may have an effect on very late stent thrombosis, and we look forward to future analyses focused on long‐term outcomes. Notably, the GLOBAL LEADERS trial did not demonstrate significant differences in ischemic outcomes between 1 and 2 years of follow‐up. A second limitation is the generalizability of these findings across all populations, especially considering the majority of these trials were based in East Asia [46, 47]. While European populations had the next‐best representation, these results may not be entirely applicable to broader populations including the US and more geographically diverse studies are needed. In light of these limitations, these results should be interpreted with caution and continue to take an individual‐based approach to antiplatelet therapies following coronary interventions. It is likely still reasonable to continue longer‐term DAPT in patients who tolerate it without complications, given that long‐term data are currently lacking. These results demonstrate, at the very least, that considering a shorter duration of DAPT after PCI is reasonable with objective evidence to support that decision.

## 8 Conclusion

In this meta‐analysis of 13 randomized trials of 53,421 patients undergoing PCI with DES, a 3‐month duration of DAPT followed by single antiplatelet therapy reduced NACE without increasing MACCE at 1 year. A 3‐month duration of DAPT followed by high potency P2Y12 may be the best performing regimen. These results are consistent in patients with and without ACS at presentation. While these results do not exclude the use of long‐term DAPT in select patients, they suggest a shortened duration of DAPT followed by a high‐potency P2Y12 inhibitor may be an acceptable alternative for most individuals, including those with normal bleeding risks (Central Illustration 1).

**Central_Illustration 1:**
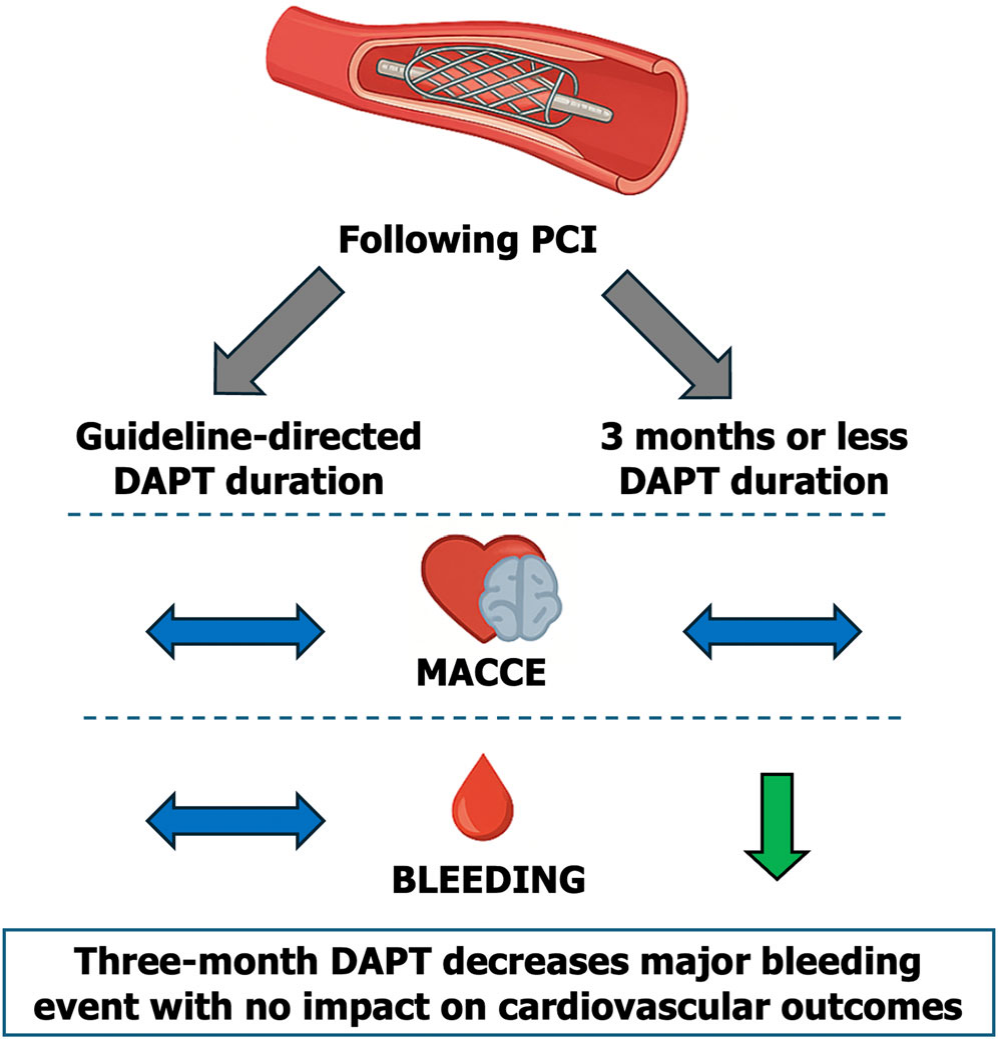
Summary of the entire cohort and relevant subgroups compared to guideline‐directed DAPT duration. [Color figure can be viewed at wileyonlinelibrary.com]

## Disclosure

All data used in this analysis is from publicly available records.

## Ethics Statement

The authors have nothing to report.

## Conflicts of Interest

The authors declare no conflicts of interest.

## Supporting information

Supplemental Figures.
